# A Compact Planar Dual-Band Multiple-Input and Multiple-Output Antenna with High Isolation for 5G and 4G Applications

**DOI:** 10.3390/mi12050544

**Published:** 2021-05-10

**Authors:** Rong Yang, Shuqi Xi, Qibo Cai, Zhizhou Chen, Xiaohang Wang, Gui Liu

**Affiliations:** 1 College of Electrical & Electronic Engineering, Wenzhou University, Wenzhou 325006, China; 184511088175@stu.wzu.edu.cn (R.Y.); 20451941038@stu.wzu.edu.cn (S.X.); caiqibo@wzu.edu.cn (Q.C.); 20170157@wzu.edu.cn (Z.C.); 2 The School of Software Engineering, South China University of Technology, Guangzhou 511442, China; xiaohangwang@scut.edu.cn

**Keywords:** multiple-input multiple-output (MIMO), 4G, 5G, dual-band antenna

## Abstract

**Featured Application:**

**The proposed dual-band MIMO antenna can be a good candidate for 5G and 4G applications.**

**Abstract:**

In this paper, a compact planar dual-band multiple-input and multiple-output (MIMO) antenna with high isolation is presented to satisfy the increasing requirements of wireless communication. The proposed antenna array consists of two identical radiating elements which are fed through micro-strip lines. A rectangular micro-strip stub with defected ground plane is employed to achieve a high isolation which is less than −15 dB between the two antenna elements. The size of the entire MIMO antenna is 32 × 32 × 1.59 mm^3^, which is printed on an FR4 substrate. The proposed MIMO antenna is optimized to operate in 2.36–2.59 GHz and 3.17–3.77 GHz bands, which can cover the fifth-generation (5G) n7 (2.5–2.57 GHz) and the fourth-generation (4G) Long Term Evolution (LTE) band 42 (3.4–3.6 GHz). The proposed MIMO antenna is feasible for the 5G and 4G applications.

## 1. Introduction

With the coming of the 5G era, it is of the utmost importance to greatly improve large-capacity data transmission and link reliability of wireless communication systems. To meet the requirements, MIMO antennas have been widely applied in wireless communication systems. However, there are many challenges in the design of MIMO antennas with compact size, high gain, and high isolation characteristics.

In recent years, various MIMO techniques for decoupling and miniaturization have been presented [1,2,3,4,5]. A tapered slot in the ground plane of a MIMO antenna provides both high isolation at microwave band and high gain at millimeter-wave band [1]. In [2], a closely coupled dual-band MIMO patch antenna with H-shaped defect ground structures achieves 34.2 dB isolation at 3.7 GHz and 36.3 dB isolation at 4.1 GHz. A novel balanced open-slot antenna as an eight-antenna MIMO array antenna element is proposed [3]. The eight-antenna array can achieve high isolation (>17.5 dB) and high total efficiency (>60%) simultaneously. A defected ground structure (DGS) and electromagnetic bandgap (EBG) are employed to reduce the mutual coupling, and the presented antenna can realize an ECC lower than 0.002 [4]. By placing a split EBG structure between two meander-line antennas, the mutual coupling can be significantly reduced [5].

Additionally, to cover as many wireless communication standards as possible, MIMO antennas with compact size, broadband, and multiband characteristics are preferred [6,7,8,9,10,11,12,13,14,15]. In [16], the presented antenna can operate in the 900 MHz, 1800–1900 MHz, 700 MHz, and 2.45 GHz bands. Similarly, in [17], a multiband and dual-element diversity antenna system with an overall size of 105 × 55 × 1.5 mm^3^ can cover an exceptionally broad bandwidth, from 890 MHz to 6 GHz. In addition, a wideband printed dual-antenna [18] with three neutralization lines can cover the GSM1800, GSM1900, UMTS, LTE2300, LTE2500, and 2.4-GHz WLAN bands. There are many MIMO antennas that can cover 5G spectrum [19,20,21]. In [22], an eight element MIMO antenna system is proposed for sub-6 GHz 5G mobile terminals. The proposed antenna array in [23] consists of an L-shaped feeding strip, a parasitic rectangle strip, and a modified Z-shaped radiating strip, which operates on the 3.5 GHz band (3.4–3.6 GHz) and 5 GHz band (4.8–5 GHz) for the applications of 5G. A multi-band MIMO antenna is designed to meet the requirements of the 4G and 5G mobile terminals with essential bandwidth for higher data rate applications [24].

In this paper, the proposed MIMO antenna has two identical antenna elements which are perpendicular to each other and a rectangular micro-strip stub is connected to the defected ground plane. The measured results show that the presented antenna can cover both 4G (3.4–3.6 GHz) and 5G (2.5–2.57 GHz) spectrums. The isolation is less than −15 dB in the desired frequency bands. The overall size of the presented compact planar dual-band MIMO antenna is 32 × 32 × 1.59 mm^3^.

## 2. Antenna Geometry and Design Consideration

**Figure 1:**
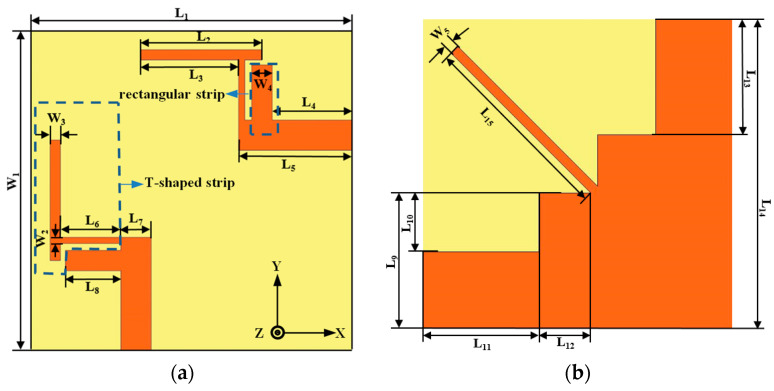
Configuration of the proposed antenna (**a**) top view; (**b**) bottom view.

**Figure 2:**
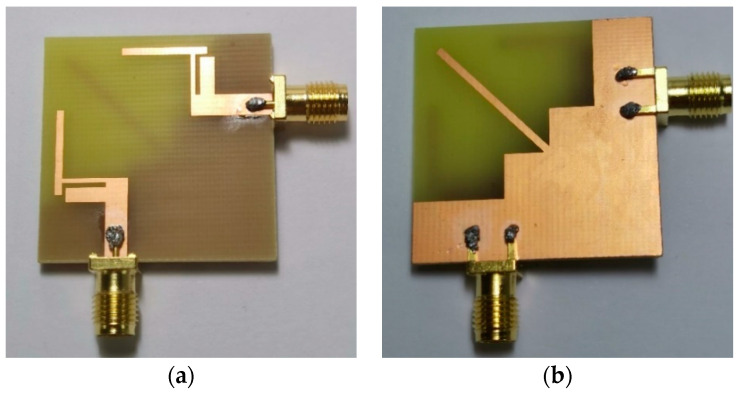
Photograph of the fabricated antenna prototype (**a**) top view; (**b**) bottom view.

The geometry of the proposed dual-band MIMO antenna is shown in Figure 1. The proposed antenna structure consists of two identical radiating elements which are fed through micro-strip lines. The antenna is fabricated on an FR4 substrate with relative permittivity ε_r_ of 4.4, loss tanδ of 0.02, and thickness of 1.59 mm. A photograph of the manufactured antenna is shown in Figure 2.

The two identical antenna elements are printed on two adjacent sides of the substrate. The radiating element is composed of a T-shaped strip and a rectangular strip. The upper half of the T-shaped strip, the lower half of the T-shaped strip, and the rectangular strip mainly realize impedance matching of the lower and higher frequency bands.

**Figure 3:**
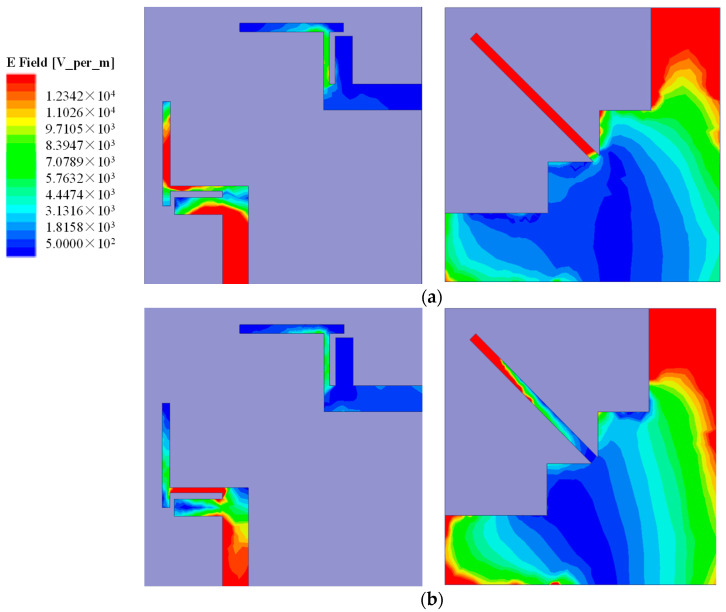
Surface current distributions at (**a**) 2.475 GHz; (**b**) 3.47 GHz.

To investigate the influence of the critical parameters of the proposed antenna, a parametric study has been carried out by changing the values of antenna dimensions. The current density distributions of the presented antenna in different frequency bands are shown in Figure 3. When the antenna operates at 2.475 GHz, the current distributions mainly appear on the upper half of the T-shaped strip and the whole rectangular strip on the bottom side as shown in Figure 3a. When the antenna operates at 3.47 GHz, the current distributions mainly appear on the lower half of the T-shaped strip and the edge of the rectangular strip on the top side of the substrate. The current only concentrates on the top part of the stub on the bottom side of the substrate, as shown in Figure 3b. Therefore, we can adjust the dimension of the T-shaped strip to optimize the current distributions which can be used to tune both resonance frequencies and isolation.

**Figure 4:**
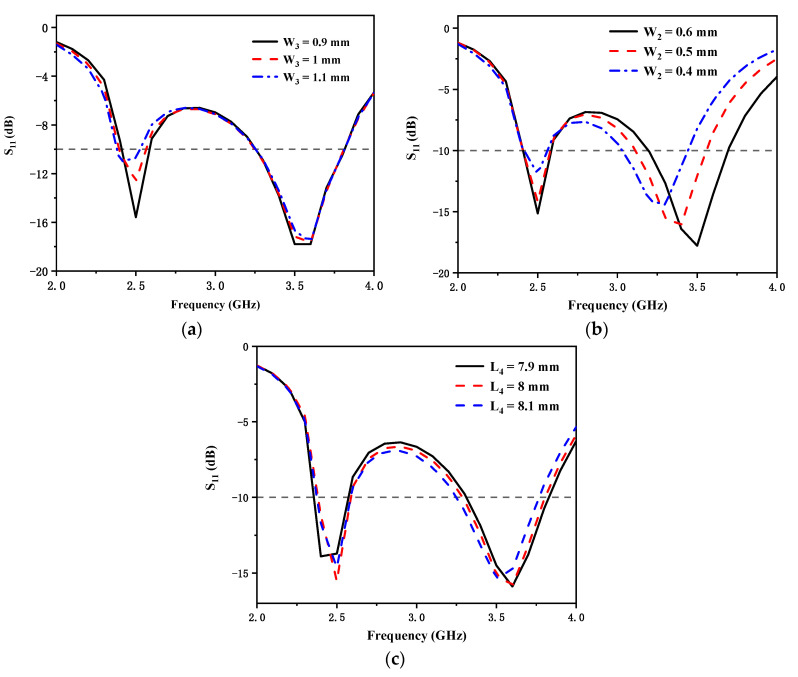
Simulated reflection coefficients for different values of (**a**) W_3_; (**b**) W_2_; (**c**) L_4_.

Figure 4 illustrates the reflection coefficients of the presented antenna with different values of W_3_ and W_2_, respectively. In Figure 4a, when the value of W_2_ is 0.5 mm, with the width of W_3_ increasing, the low-frequency mode shifts to the lower frequencies. In Figure 4b, when the value of W_3_ is 1 mm, with the width of W_2_ decreasing, the high-frequency mode shifts to the lower frequencies. In Figure 4c, when the value of W_3_ is 1 mm and W_2_ is 0.5 mm, with the width of L_4_ increasing, the high-frequency mode shifts to the lower frequencies. The optimum values of W_3_, W_2_, and L_4_ are 1 mm, 0.5 mm and 0.8 mm, respectively. The final dimensions of the proposed antenna are listed in Table 1.

## 3. Results and Discussion

**Figure 5:**
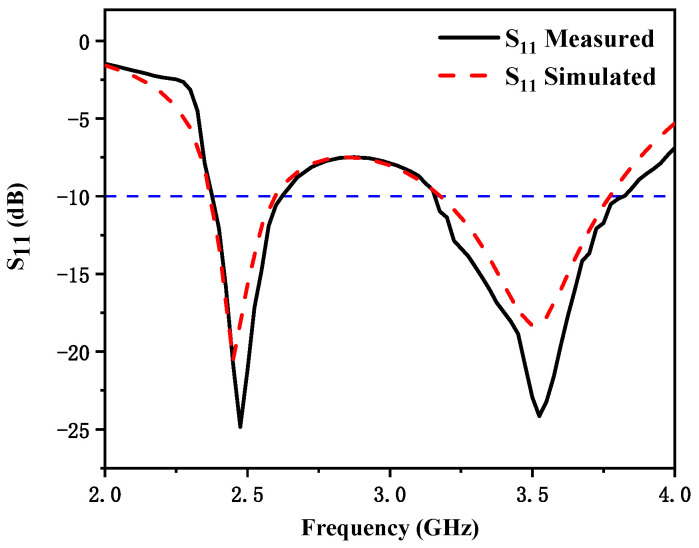
Simulated and measured S_11_ of the proposed antenna.

**Figure 6:**
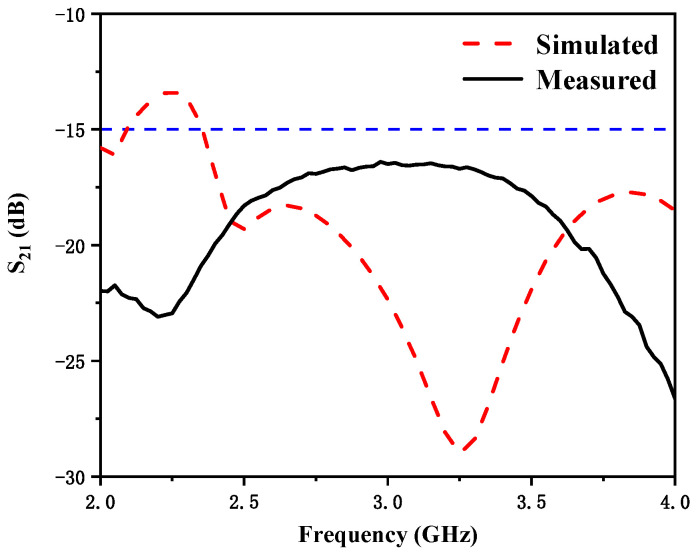
Simulated and measured S_21_ of the proposed antenna.

Figure 5 shows the simulated and measured reflection coefficients. There was a little error between the measurement results and the simulation results, which could be due to many different reasons. From the measurement results, we can see that the bandwidth with reflection coefficient less than −10 dB was 230 MHz (2.36–2.59 GHz) and 600 MHz (3.17–3.77 GHz). The realized two frequency bands can cover 5G n7 (2.5–2.57 GHz) and 4G 42 (3.4–3.6 GHz). Figure 6 shows the simulated and measured S_21_. The measured mutual coupling between the two antenna elements was less than −15 dB in the desired frequency bands. Some discrepancy between the simulated and measured results should be mainly owing to fabrication tolerance, the uncertainties of dielectric constant and loss tangent of the substrate material, and SMA connector hand soldering. Furthermore, the fabrication error may have evoked another surface current path which changed the isolation.

**Figure 7:**
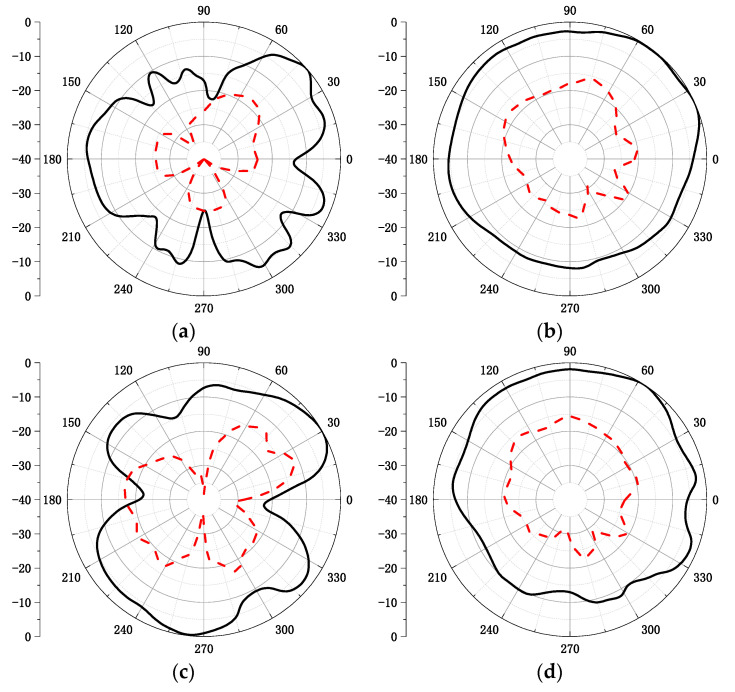
Measured radiation patterns of the proposed antenna (**a**) 2.475 GHz E-plane; (**b**) 2.475 GHz H-plane; (**c**) 3.47 GHz E-plane; and (**d**) 3.47 GHz H-plane.

**Figure 8:**
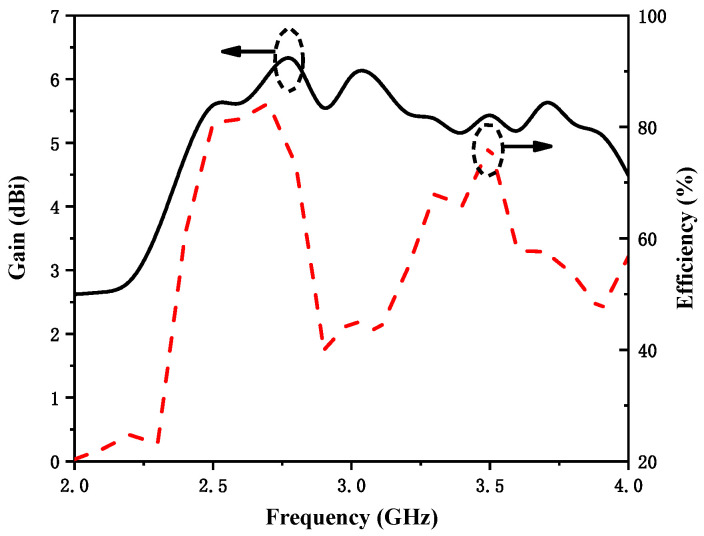
Measured antenna peak gain and total efficiency.

**Figure 9:**
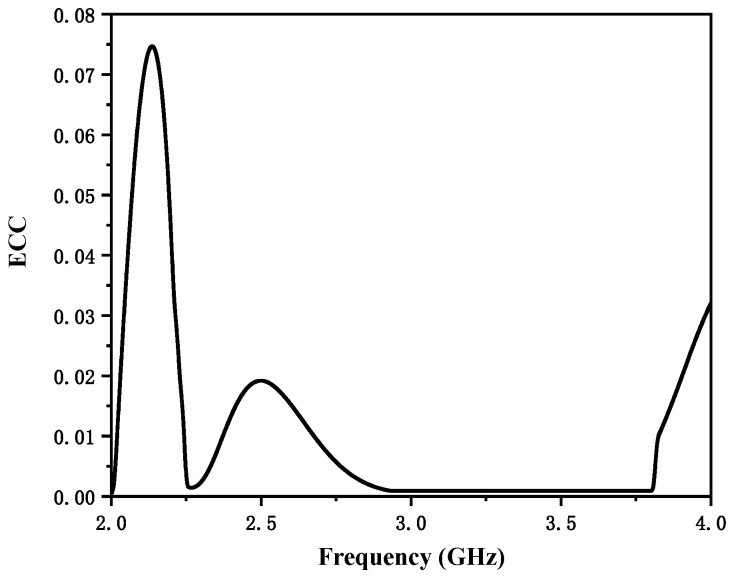
Simulated envelope correlation coefficient (ECC) of the multiple-input and multiple-output (MIMO) antenna.

Figure 7 shows the normalized measured antenna radiation patterns at 2.475 GHz and 3.47 GHz. Since the proposed antenna structure is symmetrical, one port was fixed and the other port was connected to a 50 Ω load during measurement. The solid and dash lines represent the co-polarization and cross-polarization, respectively. The measured antenna peak gain and total efficiency are presented in Figure 8. Since the radiation efficiency of an antenna is proportional to the dimension of antenna, the radiation efficiency of the lower frequency band is higher than that of the higher frequency band. The measured results show that the proposed antenna had a peak gain of 5.8 dBi at 2.36–2.59 GHz and 5.9 dBi at 3.17–3.77 GHz, and the proposed antenna had high efficiency of 80% at 2.36–2.59 GHz and 76% at 3.17–3.77 GHz. The envelope correlation coefficient (ECC) is an important indicator to judge the performance of a MIMO communication system. Figure 9 shows the simulated ECC of the MIMO antenna. The ECC was less than 0.02 over the operating band, which is acceptable for MIMO applications. A comparison of the proposed antenna with other referenced antennas is provided in Table 2. In this table, it can be observed that the achieved peak gain of the proposed antenna was higher than that of the designs demonstrated in [11,12,15]. Additionally, the size of the proposed antenna is more compact compared to other referenced antennas.

## 4. Conclusions

In this paper, a planar dual-band MIMO antenna with high isolation is presented. The two identical antenna elements were printed on two adjacent sides of the substrate. The radiating element was composed of a T-shaped strip and a rectangular strip. The proposed MIMO antenna was optimized to operate in the 2.475 GHz (2.36–2.59 GHz) and 3.47 GHz (3.17–3.77 GHz) bands, which can cover 5G n7 (2.5–2.57 GHz) and 4G LTE band 42 (3.4–3.6 GHz). The proposed antenna had a peak gain of 5.8 dBi at 2.36–2.59 GHz and 5.9 dBi at 3.17–3.77 GHz. The measured efficiency of the proposed antenna was 80% at 2.36–2.59 GHz and 76% at 3.17–3.77 GHz, respectively. The simulated ECC was less than 0.02. The proposed dual-band MIMO antenna is a good candidate for 4G and 5G applications.

## Figures and Tables

**Table 1:** Dimensions of the proposed antenna (unit: mm).

Parameters	Value	Parameters	Value	Parameters	Value
L_1_	32	L_8_	5.5	L_15_	20
L_2_	12	L_9_	14	W_1_	32
L_3_	9.7	L_10_	6	W_2_	0.5
L_4_	8	L_11_	12	W_3_	1
L_5_	11.3	L_12_	5.3	W_4_	2
L_6_	6	L_13_	12	W_5_	1
L_7_	3	L_14_	32		

**Table 2:** Comparison of the proposed antenna with other referenced antennas.

Ref.	Center Frequency (GHz)	Peak Gain (dBi)	Isolation (dB)	ECC	Total Efficiency (%)	Size (mm^2^)
[3]	3.5	-	>17.5	<0.05	76	150 × 80
[10]	5.8	6	>17	<0.05	80	50 × 80
[11]	3.6	4.3	>12	<0.03	73	80 × 40
[12]	2.44 and 5.5	2.3	>15	-	46	40 × 40
[15]	2.45 and 5.5	4.5	>17.8	<0.011	65.9	50 × 50
This work	2.475 and 3.47	5.9	>15	<0.02	81	32 × 32

## Data Availability

Data is contained within the article.
